# Double- versus single-balloon catheters for labour induction and cervical ripening: a meta-analysis

**DOI:** 10.1186/s12884-019-2491-4

**Published:** 2019-10-16

**Authors:** Xiyao Liu, Yu Wang, Fan Zhang, Xiaoni Zhong, Rong Ou, Xin Luo, Hongbo Qi

**Affiliations:** 1 Department of Obstetrics and Gynecology, The First Affiliated Hospital of Chongqing Medical University, No. 1 Youyi Road, Yuzhong District, Chongqing, 400016 China; 2 First Clinical Institute, Chongqing Medical University, No. 1 Yixueyuan Road, Yuzhong District, Chongqing, 400016 China; 3 School of Public Health and Management, Chongqing Medical University, No. 1 Yixueyuan Road, Yuzhong District, Chongqing, 400016 China

**Keywords:** Labour induction, Cervical ripening, Balloon catheter, Meta-analysis

## Abstract

**Background:**

The induction of labour is an increasingly common procedure in the obstetrics field. Various methods have been used to induce labour, among which balloon catheters play an important role. Whether the specifically designed double-balloon catheter is better than the single-balloon device in terms of efficacy, efficiency, safety and patient satisfaction remains controversial. Identifying even small differences between these two devices could be useful to guide clinical practices, to further explore their mechanisms, and to promote a better understanding of the optimal methods for inducing labour.

**Methods:**

Using the population, intervention, comparison, outcomes and study designs (PICOS) principle, we searched the PubMed, EMBASE, OVID, SCI, CENTRAL, ClinicalTrial.gov, and CDSR databases to identify relevant randomised controlled trials (RCTs) from inception through February 14, 2018. The primary outcome was the caesarean delivery rate, and the secondary outcomes focused on efficacy, efficiency, safety, and patient satisfaction. The relative risks or mean differences, including their 95% confidence intervals, were calculated using fixed-effects or random-effects models. All statistical analyses were completed with RevMan version 5.3.

**Results:**

From a total of 1326 articles, 7 RCTs involving 1159 women were included. There were no significant differences in primary outcomes (RR, 0.88 [0.65, 1.2]; *p*-value, 0.43) or secondary outcomes identified between single- and double-balloon catheters. However, heterogeneity existed for some aspects.

**Conclusion:**

Both kinds of balloon catheter have similar levels of efficacy, efficiency, safety and patient satisfaction; however, the single-balloon method is considered to be more cost-effective.

## Background

Labour induction refers to the process of artificially stimulating the uterus to begin labour [1], which is an increasingly common procedure. Cervical status, measured by the Bishop score [2], is a good predictor for the outcome of labour induction. If the cervix is unfavourable, no method is highly successful, and a ripening process is generally employed to obtain cervical effacement and dilatation prior to induction [3–5]. Methods used for cervical ripening can be broadly divided into mechanical devices and pharmacologic options [6, 7]. Compared with pharmacologic agents, mechanical methods, which were the first methods developed to ripen the cervix or induce labour [8], have similar levels of effectiveness but incur fewer episodes of adverse events (such as uterine tachysystole), have lower costs and are easier to preserve [6].

The balloon catheter, including both double- and single-balloon catheters, appears to be a widely accepted mechanical method and is recommended by the WHO for the induction process [9]. The original version of the Foley (single-balloon) catheter was initially described by Barnes in 1863 but was not described again until 1967, by Embrey and Mollison [10]. In 1991, Atad described the first double-balloon variation [11]. The Cook Cervical Ripening Balloon (CCRB), which uses an identical mechanism to that of the Atad catheter, was approved by the United States Food and Drug Administration (USFDA) in 2013 [12]. Only the double-balloon catheter (either Atad or Cook) is specifically designed and licensed for labour induction, while the Foley catheter is used beyond instructions.

Mechanical ripening devices apply pressure to the internal face of the cervix, directly overstretching the lower uterine segment and indirectly increasing the localised secretion of prostaglandin [13]. In addition to the local effect, mechanisms that involve neuroendocrine reflexes (such as the Ferguson reflex) may promote the onset of contractions [14]. Purportedly, the double-balloon (either Atad or Cook) option has an additional cervico-vaginal balloon, which applies greater pressure to both sides of the cervix and avoids the need for traction [11].

Given the increasing induction rate, the knowledge of even small differences between methods could be useful, not only to guide clinical practices but also to further explore the mechanism underlying the mechanical induction of labour and may promote a better understanding of the optimal methods for labour induction. However, studies examining the superiority of the double-balloon catheter reveal mixed results [15–23]. We conducted this meta-analysis and systematic review using the best available evidence to assess the efficacy, efficiency, safety and patient satisfaction of double-balloon catheters in comparison with those of single-balloon devices among women who underwent labour induction with unfavourable cervixes.

## Methods

### Search strategy

Together with a clinical librarian (R.O.), an electronic literature search was conducted with the PubMed, EMBASE, OVID, SCI (via WOS), CENTRAL (The Cochrane Central Register of Controlled Trials) and ClinicalTrial.gov databases from inception through ebruary 14, 2018. The searching strategy was based on the PICOS principle, utilising medical subject headings and Boolean logic-based free-text combinations of the following search items: “labour induction”, “cervical ripening”, “balloon”, “Foley”, “Cook” and “Atad”. In addition, we used sensitivity-maximising search filters to identify randomised controlled trials [24]. With the abovementioned databases, several meta-analyses and systematic reviews were identified. Aiming to identify more pertinent meta-analyses or systematic reviews, an additional search was performed in the CDSR (Cochrane Database of Systematic Reviews) database. All of the reference lists from the relevant reviews were manually retrieved to locate further eligible trials. There were no language restrictions. Differences of opinion were resolved by team discussion.

### Study selection and data collection

All related RCTs that directly compared the double-balloon catheter with the single-balloon catheter for the purposes of labour induction or cervical ripening were included in the analysis. There were no restrictions with regards to settings, demographics, obstetrics characteristics (e.g., race, maternal age, and gestational weeks) and outcome measures. We excluded the following types of studies: (1) studies of balloon catheters used for outpatient purposes; and (2) protocols, observational studies, and secondary analyses of previous studies and guidelines. Prior to the formal review process, we performed pretesting with the kappa statistic to calculate the level of agreement between the inclusion/exclusion decisions of different reviewers and adjusted our criterion until kappa ≧ 0.75.

To improve the precision of the collected data, two reviewers (X.Y.L., Y.W.), one who majored in obstetrics and one who did not, screened each record for eligibility and independently extracted and tabulated the following information from the text, tables, and graphs: lead author; publication year; country of origin; study design; participants and intervention characteristics; outcomes; and sponsor. Prior to determining the categories for the data collection forms, a pilot test was performed using representative samples of the studies to be reviewed. All of the collected data are available upon request.

Due to the uncertain benefits of blinded assessments and the large workload, we did not conceal the general contents of the studies during this process. Any disagreements were resolved through discussion, or if necessary, through consultation with a third reviewer (F.Z.) who specialises in evidence-based medicine. When information regarding any of the extracted data points listed above was unclear, an attempt was made to access further details by contacting the authors of the original reports.

### Selection of outcomes

The primary and secondary outcomes were defined before trial retrieval was performed. The primary outcome was the caesarean delivery rate. The secondary outcomes included: (1) catheter placement (placement difficulty/failure, spontaneous expulsion); (2) intervals (insertion to delivery, insertion to expulsion/removal, expulsion to delivery); (3) Bishop score increment; (4) vaginal delivery (vaginal delivery within 24 h, normal vaginal delivery, assisted vaginal delivery); (5) analgesia usage; (6) maternal adverse events (death, infection, postpartum haemorrhage); (7) neonatal adverse events (death, low Apgar score, NICU admission); (8) length of hospitalisation; and (9) satisfaction **(**pain during the process, maternal total satisfaction). While we attempted to collect all of the above datapoints from all of the analysed studies, only those that provided all of the data appear in the analysis tables.

### Quality assessment

Two independent investigators (X.Y.L., Y.W.) openly (not blinded) assessed the methodological quality of the included RCTs based on Cochrane risk-of-bias tool. Quality was graded based on the following criteria [25]: (1) high quality: both randomisation and allocation concealment were assessed as having low risks of bias, and all other items were assessed as having low or unclear levels of risk; (2) low quality: either randomisation or allocation concealment was assessed as having a high risk of bias, regardless of the risk levels of other items; and (3) moderate quality: trials did not meet the criteria for high or low quality. Discrepancies were resolved by consensus.

### Statistical analysis

All statistical analyses were performed with RevMan version 5.3, with the help of a statistician (X.N.Z.). The relative risks (RRs) and mean differences (MDs), with corresponding 95% confidence intervals (CIs), were used to describe the intervention effects for dichotomous and continuous variables, respectively. All potential data conversions utilised standard formulae recommended by the Cochrane Handbook [24].

Heterogeneity was identified by Cochrane’s Q test and the I^2^-statistic test, in which a Q test *p*-value < 0.1 and an I^2^ value ≥50% indicated significant heterogeneity. When both the *p*-value and the I^2^ value displayed no heterogeneity, we chose the fixed-effect model. Else, a random-effect model was used.

Subgroup analysis was pre-specified and performed on parity. A sensitivity analysis was conducted to identify studies involving data conversions that may have exerted a disproportionate influence on the pooled estimates. We assessed publication bias by examining funnel plots for the primary outcome only.

## Results

### Study characteristics

**Fig. 1:**
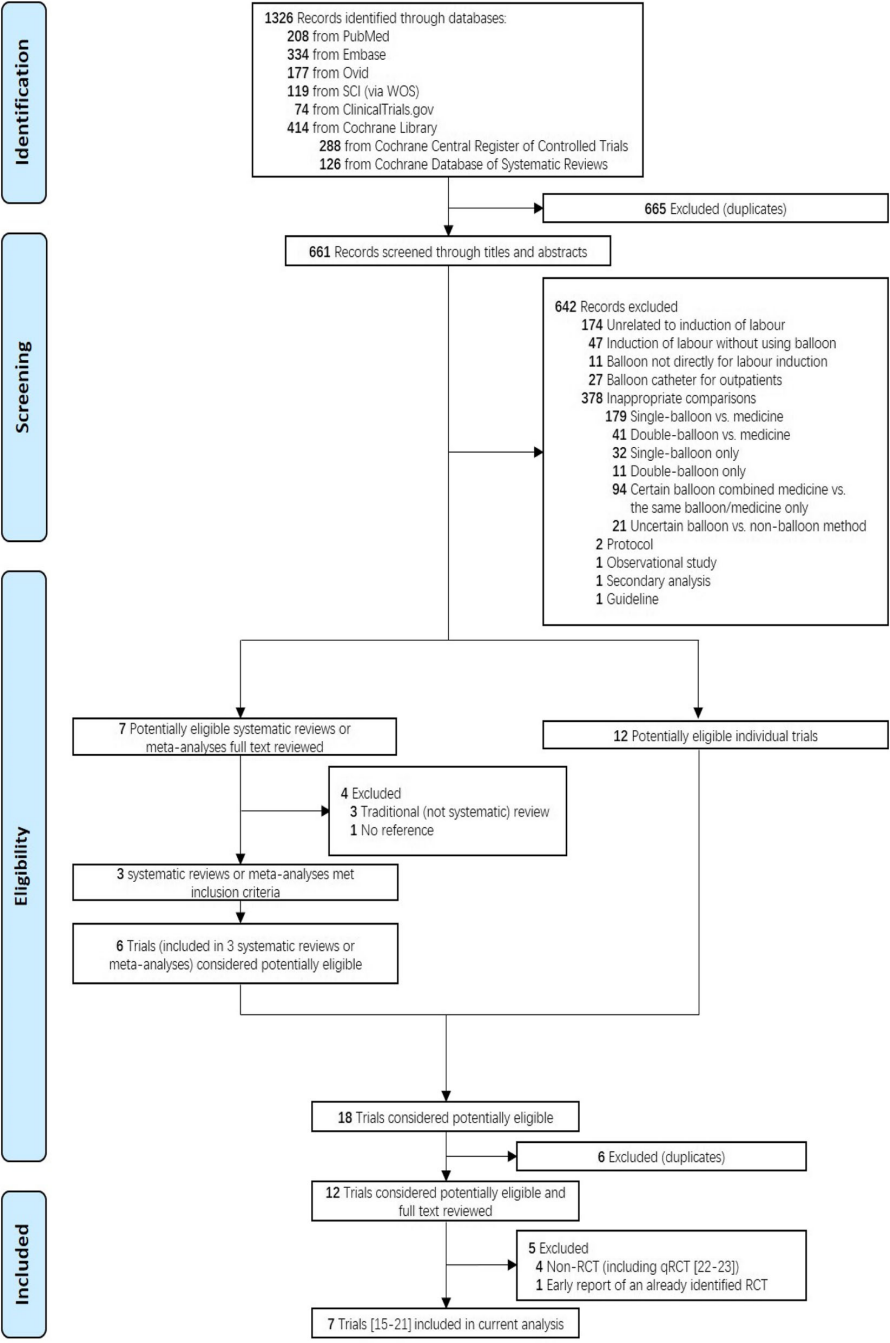
Literature search and screening process

**Fig. 2:**
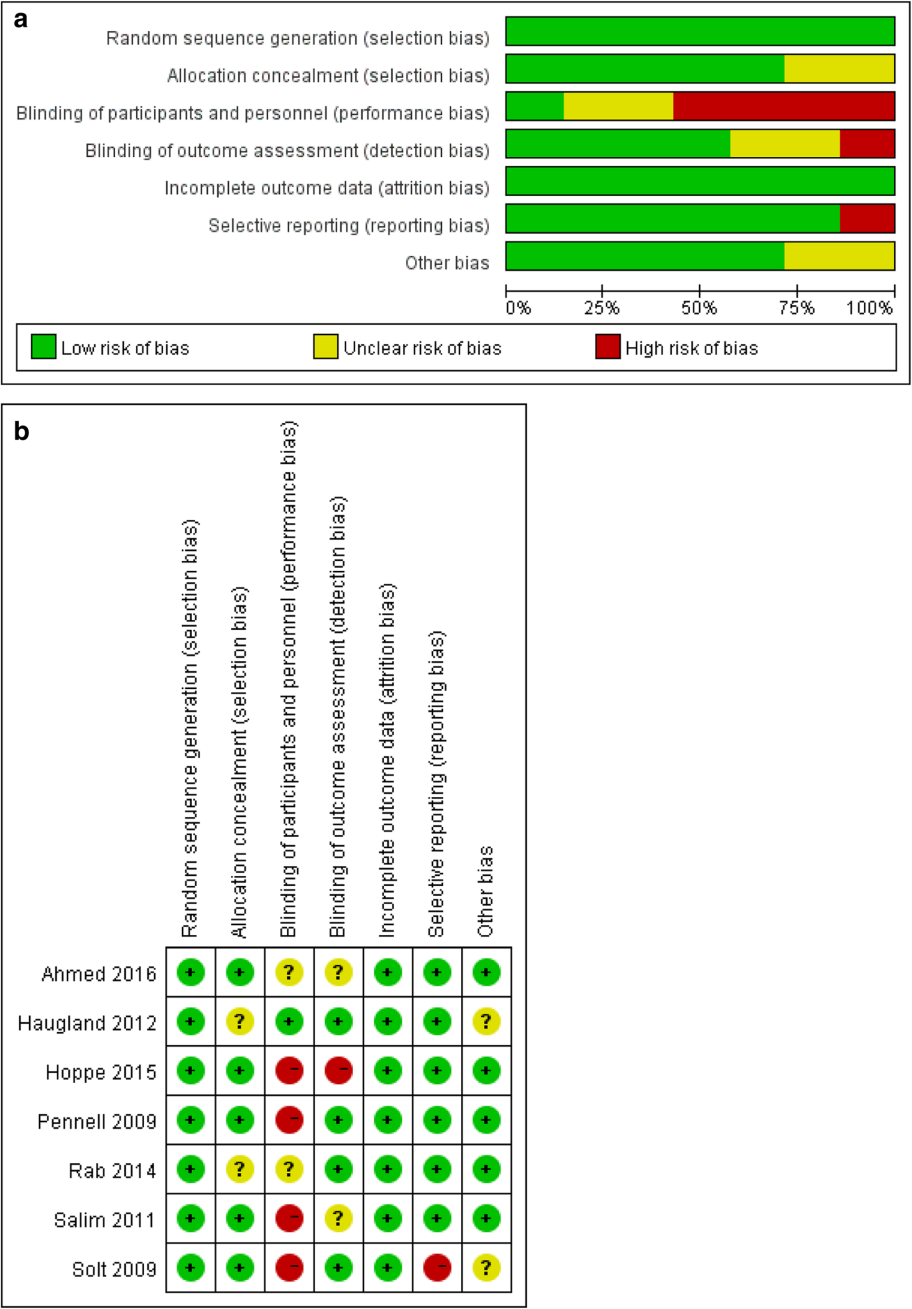
**a** Risk of bias graph. **b**. Risk of bias summary

The literature search and screening process is shown in Fig. 1. Initially, 1326 potentially relevant records were identified. The titles and abstracts were reviewed, and 12 relevant trials were further screened. After thorough investigation, 7 RCTs, containing 1159 women and available data (577 and 582 in the double- and single-balloon groups, respectively), were determined to be eligible for inclusion [15–21]. The characteristics of the included trials are summarised in Table 1. Table 2 shows the risk of bias and the corresponding quality of each individual trial, which is illustrated in Fig. 2a and b. Basic demographic and obstetric variables are presented in Table 3. Except for postdates, which only two studies reported and which show slight heterogeneity, all other variables were comparable.

**Table 1:** Characteristics of the included trials

Study	Period	Country	Method	Sample size total (double/single)	Parity total (double/single)	Balloon Catheter	
						Double^x^	Single^y^
Ahmed 2016	2013.03–2014.04	Egypt	RCT	78 (39/39)	all nulliparous	Cook	Foley (50 ml)
Haugland 2012	2010.03–2011.01	Norway	RCT	178 (88/90)	NA	Cook	Foley (NA)
Hoppe 2015	2010.01–2013.11	USA	RCT	98 (50/48)	nulliparous: 50 (25/25) multiparous: 48 (25/23)	Cook	Foley (30 ml)
Pennell 2009*	2001.07–2003.12	Australia	RCT	217 (107/110)	all nulliparous	Atad	Foley (30 ml)
Rab 2015	2011.01–2013.12	Egypt	RCT	200 (100/100)	nulliparous: 113 (55/58) multiparous: 87 (45/42)	Cook	Foley (30 ml)
Salim 2011	2008.06–2010.12	Israel	RCT	293 (148/145)	nulliparous: 155 (78/77) multiparous: 138 (70/68)	Cook	Foley (60 ml)
Solt 2009	2006.01–2008.05	Israel	RCT	95 (45/50)^§^	nulliparous: 95 (45/50) multiparous: 85 (NA)^§^	Cook	Foley (NA)

Except for two studies [16, 21], in which we could not find detailed information, all studies offered similar standard instructions for how to use the balloon catheters

x: COOK/Atad: 80 ml + 80 ml, without tension

y: All Foley catheters were applied with light tension

NA: Data not found: unable to contact the authors of the original reports

*: Pennell 2009 [18] was a multi-arm study, and we only extracted data for the double-balloon catheter and single-balloon catheter comparison arms

§: Solt 2009 [21] only reported the results of nulliparous women; therefore, we eliminated the multiparous subgroup and extracted nulliparous data only.

**Table 2:** Risk of bias and corresponding quality

Study	Risk of bias for the included studies							Quality
	Random sequence generation (selection bias)	Allocation concealment (selection bias)	Blinding of participants and personnel (performance bias)^x^	Blinding of outcome assessment (detection bias)	Incomplete outcome data (attrition bias)	Selective reporting (reporting bias)^y^	Other bias	
Ahmed 2016	L	L	U	U	L	L	L	H
Haugland 2012	L	U	L	L	L	L	U	M
Hoppe 2015^A^	L	L	H	H	L	L	L	M
Pennell 2009	L	L	H	L	L	L	L	H
Rab 2014	L	U	U	L	L	L	L	M
Salim 2011^A^	L	L	H	U	L	L	L	H
Solt 2009^B^	L	L	H	L	L	H	U	M

Other bias: trials sponsored by drug companies or trials in which baseline characteristics were not similar between different intervention groups

L: Low risk or low quality. For the risk of bias, L means appropriate methods were adequately described

H: High risk or high quality. For the risk of bias, H means high risk was found (unable to avoid bias)

U: Unclear risk, no description

M: Moderate quality

A: Hoppe et al. [17] and Salim et al. [20] reported a few lost follow-ups and did not perform intention-to-treat (ITT) analyses. Fortunately, they were balanced in numbers with similar reasons across intervention groups and had little influence on the following analysis. Therefore, we assessed the attrition bias as low

B: Solt et al. [21] only described a single-blind method. Considering the nature of the study, we evaluated performance bias as H, while detection bias was evaluated as L. Additionally, they selectively reported outcomes for the nulliparous group, and we could not obtain supplemental data for the multiparous group by contacting the author

x: Blinding of participants and personnel, though graded, was excluded from the quality assessment because it was impractical for these trials

y: Some studies did not offer their protocols; therefore, it is difficult hard to determine whether the outcomes were not measured or not reported. Unless selective reporting was obvious, we evaluated this situation as being low risk

**Table 3:** Basic demographic and obstetric variables

Variables*	Number of studies analysed	Interventions		Pooled effect (95% CI)^x^	Q *p*-value^y^	I^2^- statistic^y^	*p*-value^z^
		Double	Single				
Maternal age (years)^A^	5 [15,17–20]	444	442	0.73 [−0.08, 1.53]	0.89	0	0.08
Gestational weeks (weeks)^A^	5 [15,17–20]	444	442	−0.07 [− 0.31, 0.17]	0.71	0	0.56
Bishop score at catheter insertion^B^	4 [15,17,19–20]	337	332	0.02 [−0.19, 0.23]	0.42	0	0.85
Induction indications							
Postdates^C^	2 [17–18]	157	158	1.10 [0.51, 2.37]	0.15	**51%**	0.81
Diabetes mellitus	3 [17–18,20]	305	303	1.35 [0.80, 2.26]	0.20	39%	0.26
Hypertensive disease	3 [17–18,20]	305	303	0.98 [0.66, 1.44]	0.21	35%	0.91
Intrauterine growth restriction	2 [17–18]	157	158	0.92 [0.39, 2.21]	0.23	29%	0.86

*: Only those variables contained in more than one study are displayed in this analysis table

Only Hoppe 2015 [17] described oligohydramnios and abnormal foetal monitoring and did not reveal significant differences

Rab 2014 [19] mentioned data on body mass index (BMI) at insertion, while Salim, 2011 [20] reported BMI data before pregnancy

Not all of the baseline data was described in Haugland, 2012 [16] and Solt, 2009 [21]

A: Pennell 2009 [18] described the maternal age and gestational weeks by median and typical range. The results remained the same after sensitivity analysis

B: Pennell 2009 [18] described the Bishop score using ordinal data, making synthesis impossible

C: Postdates, as one of the induction indications, shows heterogeneity between the two studies [17, 18]

x: The odds ratio (OR) was the pooled effect for dichotomous variables. The mean difference (MD) was the pooled effect for continuous outcomes

y: Study heterogeneity was explored using Cochrane’s Q test and the I^2^-statistic test. Random- and fixed-effect models were used as appropriate

z: The *p*-value ≥0.05 for the test indicated that the pooled effect was insignificant; for dichotomous outcomes, the test was OR = 1; for continuous outcomes, the test was MD = 0

Of the 7 RCTs, 3 trials [15, 18, 21] focused on nulliparous women, while 2 trials [17, 20] conducted subgroup analysis by parity. These 5 trials, which included 781 women (595 nulliparous and 186 multiparous), were suitable for parity subgroup analysis.

### Effects of interventions

**Fig. 3:**
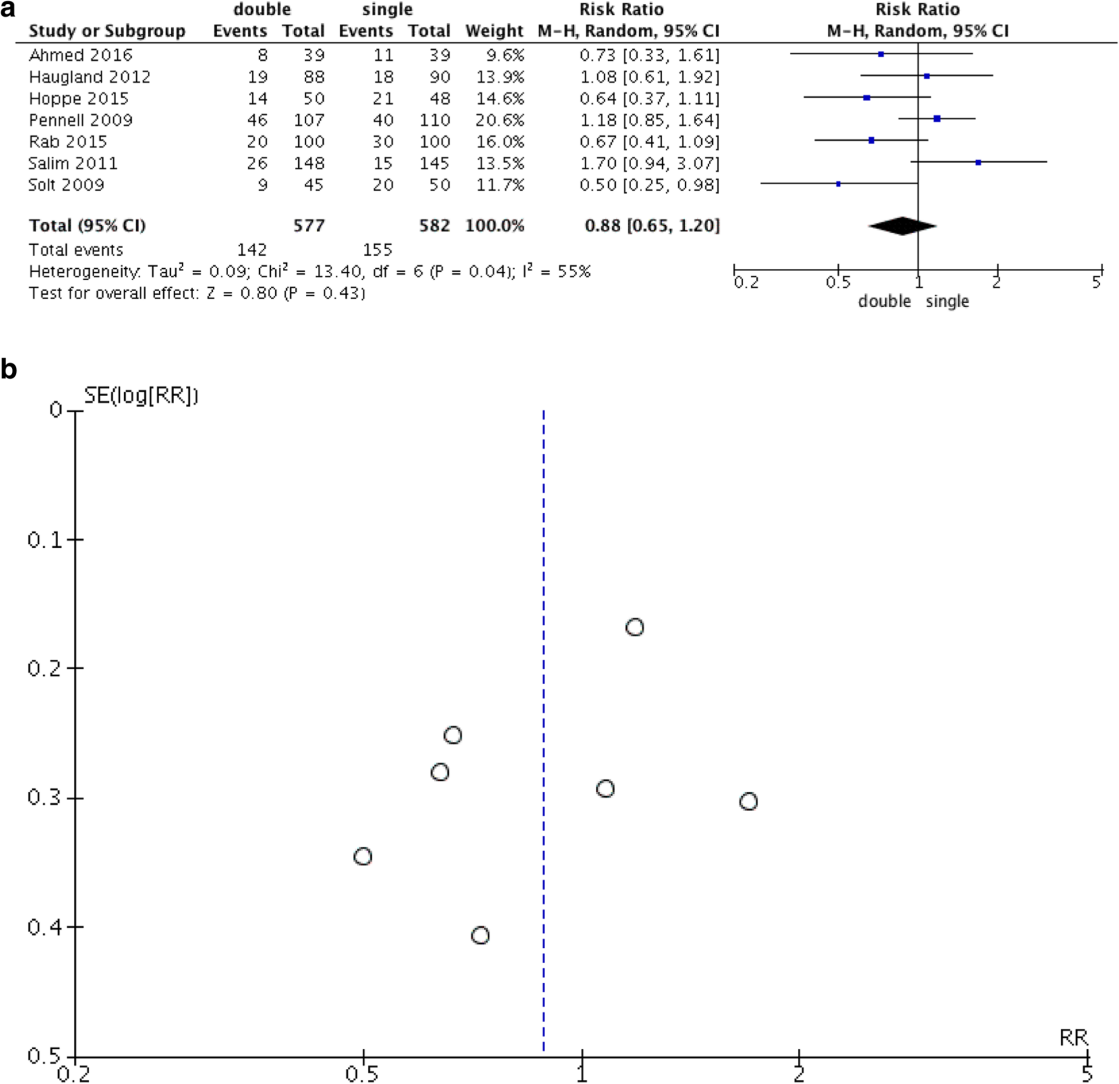
**a** Forest plot of cesarean delivery. **b**. Funnel plot of cesarean delivery

All trials reported the rates of caesarean section. There were no significant differences in the rates of caesarean delivery (RR, 0.88 [0.65, 1.2]; *p*-value, 0.43) among trials, but heterogeneity existed (Q *p*-value, 0.04; I^2^, 55%) (Fig. 3a). A corresponding funnel plot is shown in Fig. 3b. During sensitivity analysis, heterogeneity disappeared only when Salim 2011 [20] was excluded (Q *p*-value, 0.11; I^2^, 45%), while the pooled effect was always robust (no significant differences). The secondary outcomes, shown in Table 4, did not differ obviously between the two types of catheter, except for the Bishop score increment (MD, 0.57 [0.28, 0.86]; *p*-value, 0.0001).

**Table 4:** Secondary outcomes

Outcomes	Number of studies analysed	Interventions		Effect measure	Pooled effect (95% CI)	Q *p*-value	I^2^- statistic	*p*-value	Sensitivity analysis
		Double	Single						
placement difficulty/failure	4 [15–16,18,20]	382	384	RR	1.34 [0.66, 2.71]	0.55	0	0.42	stable
spontaneous expulsion	4 [15,17,19–20]	337	332	RR	0.86 [0.60, 1.23]	**0.0002**	**85%**	0.40	stable
insertion to delivery interval^A^	6 [15–20]	532	532	MD	0.98 [− 0.03, 2.00]	0.16	36%	0.06	**Unstable** ^I^
					1.36 [−0.63, 3.34]	**0.02**	**61%**	0.18	**Unstable** ^II^
insertion to expulsion/removal interval^B^	4 [15,17,19–20]	278	267	MD	1.72 [−0.35, 3.79]	**< 0.00001**	**93%**	0.10	**Unstable** ^III^
expulsion to delivery interval	2 [19,21]	145	150	MD	−2.81 [−10.82, 5.19]	**0.06**	**72%**	0.49	–
Bishop score increment^C^	5 [15,17,19–21]	382	382	MD	0.57 [0.28, 0.86]	0.30	18%	**0.0001**	stable
vaginal delivery within 24 h	3 [17–18,20]	305	303	RR	0.95 [0.72, 1.26]	0.11	**54%**	0.74	**Unstable** ^IV^
normal vaginal delivery^D^	6 [15–20]	532	532	RR	1.02 [0.86, 1.20]	**0.03**	**59%**	0.84	**Unstable** ^V^
assisted vaginal delivery^D^	4 [16,18–20]	443	445	RR	1.08 [0.84, 1.41]	0.48	0	0.54	stable
analgesia usage	4 [17–20]	405	403	RR	1.07 [0.99, 1.16]	0.72	0	0.10	stable
maternal adverse events									
maternal infection	5 [17–21]	450	453	RR	1.04 [0.66, 1.66]	0.38	5%	0.85	stable
postpartum haemorrhage	3 [15,18–19]	246	249	RR	1.03 [0.74, 1.42]	0.73	0	0.88	stable
neonatal adverse events									
low Apgar score (< 7 at 5 min)^E^	3 [17–18,20]	305	303	RR	0.53 [0.15, 1.88]	0.46	0	0.32	–
NICU admission	3 [17–18,20]	305	303	RR	0.70 [0.45, 1.07	0.90	0	0.10	stable
length of hospitalisation	2 [19–20]	248	245	MD	0.16 [−0.10, 0.41]	0.28	15%	0.22	–
satisfaction^F^									
pain during the process	2 [15,19]	139	139	MD	0.07 [−0.53, 0.67]	0.42	0	0.82	–
maternal total satisfaction	2 [15,19]	139	139	MD	−0.10 [−1.25, 1.04]	**0.08**	**68%**	0.86	–

A: No studies, except for those of Hoppe 2015 [17] and Salim 2011 [20], specifically defined delivery as being either total delivery or vaginal delivery. Hoppe 2015 [17] offered data on the time from insertion to vaginal delivery, while Salim 2011 [20] reported both measurements. We synthesised these data by involving Hoppe 2015 [17]. The upper and lower data shows the effects when we added the total and vaginal delivery data from Salim 2011 [20]

B: Salim 2011 [20] excluded 124 women (70 in the double-balloon catheter group and 54 in the single-balloon catheter group) with spontaneous expulsion during this process

C: We depended primarily on the Bishop score increment. For those studies that included only a second Bishop score, we included these data and conducted sensitivity analyses

D: Hoppe 2015 [17] reported only vaginal deliveries but did not define whether assisted vaginal deliveries were included; we treated these data as though it did not include assisted vaginal deliveries

E: Salim 2011 [20] reported no events on this outcome for either arm, which was inestimable

F: All measured by VAS

I: When we eliminated Salim 2011 [20], the MD pooled effect changed to 2.16 [0.76, 3.57] (*p*-value, 0.003), in favour of the single-balloon catheter. The results remained comparable after all other sensitivity analyses were performed

II: Excluding Rab 2014 [19], though heterogeneity disappeared, the effect remained comparable (Q *p*-value, 0.17; I^2^, 38%; *p*-value, 0.58). Excluding Salim 2011 [20], the result was shown in superscript note I

III: Significant heterogeneity existed regardless of which study we excluded; however, when we repeated the analysis after excluding Salim 2011 [20], the result changed (MD, 2.40 [0.32, 4.48]; supporting the single-balloon catheter)

IV: Stable effect but became homogeneous only when we excluded Hoppe 2015 [17]

V: Stable effect but became homogeneous only when we excluded Rab 2014 [19] or Salim, 2011 [20]

Subgroup analysis results by parity are shown in Tables 5 and Table 6. Only the Bishop score increment in nulliparous women exhibited a statistically significant difference; however, heterogeneity was demonstrated among studies (MD, 1.08 [0.38, 1.78]; Q *p*-value, 0.11; I^2^, 56%; *p*-value, 0.002), suggesting that the double-balloon catheter may have a greater ability to increase the Bishop score. Unless otherwise **highlighted**, studies were homogeneous, and sensitivity analysis displayed no meaningful changes.

**Table 5:** Outcomes by parity (nulliparous)

Outcomes*	Number of studies analysed	Interventions		Effect measure	Pooled effect (95% CI)	Q *p*-value	I^2^- statistic	*p*-value	Sensitivity analysis
		Double	Single						
caesarean delivery	5 [15,17-18,20–21]	294	301	RR	0.86 [0.56, 1.33]	**0.02**	**65%**	0.50	stable
placement difficulty/failure	2 [15,18]	146	149	RR	0.72 [0.15, 3.55]	0.57	0	0.69	–
spontaneous expulsion	1 [15]	39	39	RR	0.88 [0.70, 1.10]	–	–	0.27	–
insertion to delivery interval^A^	4 [15,17-18,20]	249	251	MD	0.88 [− 0.43, 2.18]	0.59	0	0.19	stable
					0.43 [−0.84, 1.71]	0.26	26%	0.50	stable
insertion to expulsion/removal interval	2 [15,17]	64	64	MD	0.88 [−0.00, 1.76]	0.38	0	0.05	–
expulsion to delivery interval	1 [21]	45	50	MD	−8.00 [−16.35, 0.35]	–	–	0.06	–
Bishop score increment^C^	3 [15,17,21]	109	114	MD	1.08 [0.38, 1.78]	0.11	**56%**	**0.002**	**Unstable** ^**I**^
vaginal delivery within 24 h	3 [17–18,20]	210	212	RR	0.91 [0.75, 1.10]	0.19	40%	0.33	stable
normal vaginal delivery^D^	4 [15,17-18,20]	249	251	RR	1.00 [0.78, 1.29]	0.17	**58%**	0.98	**Unstable** ^**II**^
assisted vaginal delivery^D^	2 [18,20]	185	187	RR	1.02 [0.65, 1.59]	0.56	0	0.94	–
analgesia usage	2 [18,20]	185	187	RR	1.06 [0.95, 1.19]	0.32	1%	0.28	–
maternal adverse events									
maternal infection	3 [18,20–21]	230	237	RR	1.16 [0.69, 1.95]	0.27	24%	0.58	stable
postpartum haemorrhage	2 [15,18]	146	149	RR	1.00 [0.72, 1.40]	0.49	0	0.98	–
neonatal adverse events									
low Apgar score (< 7 at 5 min)^E^	2 [18,20]	185	187	RR	0.21 [0.01, 4.23]	–	–	0.31	–
NICU admission	1 [18]	107	110	RR	0.74 [0.45, 1.22]	–	–	0.24	–
satisfaction^F^									
pain during the process	1 [15]	39	39	MD	−0.40 [−1.69, 0.89]	–	–	0.54	–
maternal total satisfaction	1 [15]	39	39	MD	0.47 [−0.42, 1.36]	–	–	0.30	–

* Superscript notes (A-F) are the same as those for Table 4

I: Excluding Hoppe 2015 [17], although heterogeneity disappeared, the effect remained (Q *p*-value, 0.46; I^2^, 0; *p*-value, 0.002). Excluding Solt 2009 [21], heterogeneity still existed, but the effect significance disappeared (Q *p*-value, 0.03; I^2^, 78%; *p*-value, 0.07)

II: Results remained comparable, but heterogeneity disappeared after we excluded Hoppe 2015 [17] (Q *p*-value, 0.27; I^2^, 24%; *p*-value, 0.21)

**Table 6:** Outcomes by parity (multiparous)

Outcomes*	Number of studies analysed	Interventions		Effect measure	Pooled effect (95% CI)	Q *p*-value	I^2^- statistic	*p*-value	Sensitivity analysis
		Double	Single						
caesarean delivery	2 [17,20]	95	91	RR	1.35 [0.54, 3.36]	0.43	0	0.52	–
insertion to delivery interval^A^	2 [17,20]	95	91	MD	−0.48 [−2.24, 1.28]	0.62	0	0.60	–
					−1.05 [− 2.68, 0.58]	0.70	0	0.21	–
insertion to expulsion/removal interval	1 [17]	25	23	MD	2.30 [−0.97, 5.57]	–	–	0.17	–
Bishop score increment^C^	1 [17]	25	23	MD	0.20 [−0.88, 1.28]	–	–	0.72	–
vaginal delivery within 24 h	2 [17,20]	95	91	RR	1.05 [0.93, 1.18]	0.21	36%	0.46	–
normal vaginal delivery^D^	2 [17,20]	95	91	RR	0.92 [0.83, 1.02]	0.36	0	0.13	–
assisted vaginal delivery^D^	1 [20]	70	68	RR	8.75 [0.48, 159.42]	–	–	0.14	–
analgesia usage	1 [20]	70	68	RR	1.20 [0.70, 2.07]	–	–	0.51	–
maternal adverse events (infection)	1 [20]	70	68	RR	2.92 [0.12, 70.35]	–	–	0.51	–
neonatal adverse events (low Apgar score)^E^	1 [20]	70	68	RR	Not estimable^§^	–	–	–	–

* Superscript notes (A-F) are the same as those for Table 4

§: Reporting no events in both groups, which were inestimable

## Discussion

### Summary of main results

#### Efficacy and efficiency

Balloon catheters were initially designed for cervical dilatation and ripening during labour induction. The best indicator of efficacy is the Bishop score increment. However, when correlated with baseline data, the Bishop score served only as a secondary outcome. No significant differences were observed for obstetric characteristics (including the Bishop score before catheter insertion) between women treated with the single-balloon catheter and those treated with the double-balloon catheter. Therefore, we could use the Bishop score after catheter removal (the second Bishop score) to roughly calculate this effect size, and it was not necessary to perform covariance analyses to adjust the baseline data. According to our analysis, the double-balloon catheter increases the Bishop score more significantly, especially for nulliparous women. However, this result was not observed for the multiparous subgroup. In support of this finding, one study [17] reported a Bishop score > 6 at balloon removal, and a similar trend in was observed for both general and subgroup subjects. Additionally, the ripening success rates (defined by the individual articles) appeared to be higher in the double-balloon groups, but without enough statistical power to determine significance [16, 19, 22, 23]. Atad et al. also reported similarly large average increments in the Bishop scores for both nulliparous and multiparous women for the double-balloon catheter, without a single-balloon catheter comparison group [11]. Later, the researchers reported that the Bishop score increment when employing the single-balloon catheter was lower than that achieved by the double-balloon catheter, with a higher failure rate [26].

Efficiency, best evaluated by the interval length and the 24 h delivery rate, is comparable regardless of parity. In the double-balloon catheter group, the interval from insertion to delivery appears to be longer, while the interval from expulsion to delivery appears to be shorter, though neither measure achieves significance. Ahmed, et al. [15] stated that women treated with a single-balloon catheter had a shorter insertion to amniotomy time (*p* = 0.02) than women treated with a double-balloon catheter, while Pennell, et al. [18] found that the length of labour did not significantly differ (*p* = 0.152) between the two groups; there is little consensus on the time from insertion to active labour, with Pennell, et al. [18] preferring the single-balloon catheter (*p* = 0.014), while Rab, et al., [19] demonstrated no obvious differences. Ahmed and Mei-Dan [15, 22] suggested that the shorter interval between insertion and expulsion for the single-balloon catheter likely resulted in the observed shorter induction to delivery interval, although the second Bishop score was lower in this group.

The frequency of placement difficulty or failure and spontaneous expulsion are similar between the two groups. In addition, Salim, et al. [20] found that women who spontaneously expelled their catheter demonstrated favourable outcomes with regards to shorter times from induction to delivery (1.10 [1.06–1.15]; *p* = 0.001) and a significantly lower proportion of operative deliveries (2.15 [1.26–3.69]; *p* = 0.003).

#### Safety

Both maternal and neonatal adverse events are of great concern. Although we hoped to consider mortality data, no study provided this information. Other measurements were also equivalent, including maternal infection, postpartum haemorrhage, low Apgar scores and NICU admissions. Some studies also [18, 20] reported placental abruptions, uterine hyperstimulation, cord prolapse, malpresentation, and Apgar < 4 at 1 min, with no significant differences between groups.

#### Satisfaction

Patient-reported outcomes (PROs), such as maternal satisfaction, represent what is most important to patients about a condition and its treatment [24]. However, few reports related to PROs were found. Here, we can report patient satisfaction based on two original reports [15, 19], both evaluated by the visual analogue scale (VAS) [27], with identical measurement times and protocols. The pooled results of these two studies suggests similar satisfaction levels for the two catheter types.

#### Comprehensive outcomes

Delivery modes, which are of particular clinical concern, represent a comprehensive measurement of the effectiveness and safety of labour induction protocols and can incorporate economic evidence. Caesarean section delivery is the most frequently used outcome pre-specified by trials. According to our analysis, no strong evidence exists to demonstrate which mechanical device is more effective, and heterogeneity exists among studies. Similarly, both normal and assisted vaginal delivery rates were comparable between groups, regardless of parity, as were the rates of analgesia usage during the ripening process and the lengths of hospitalisation.

#### Heterogeneity

Heterogeneity exists in many results, which may be the result of differences in study design or quality, participants, interventions, demographic feature or local policies. During our heterogeneity test, three studies [17, 19, 20] were potential candidates for being the sources of heterogeneity. Unlike other studies, Rab et al. [19] enrolled women who had experienced a stillbirth and had scarred uteri, which could be responsible increasing the general heterogeneity. Additional differences among these studies involved parity and balloon volumes (discussed below).

### Applicability of evidence

#### Guide clinical Practice

Despite the fact that the double-balloon makes results in more favourable Bishop scores, it appears to result in prolonged intervals. No differences were observed for delivery mode, which is the most meaningful obstetrical outcome. As for the economical consideration, it is mainly related to hospitalization length, delivery mode and device itself. What is noteworthy is that the single balloon (Foley catheter) is approximately 30–40 times cheaper than the double-balloon catheter at different institutions, and the difference of price varies from countries to countries. As the producers offered that Foley catheters cost approximately $1.12, while Cook catheters cost approximately $39.33. As for our hospital in China, the single balloon catheter costs 20–30 RMB while the double one costs 600 RMB, and the price for placing balloon catheters is about 600 RMB in both situations. Considering the fact that caesarean section and hospitalization length were similar in the two groups, and when coupled with a substantial price differences in the devices, the single balloon catheter seems like to be more cost-effective for labour induction, particularly in low resource settings.

#### Exploring the mechanisms

Practically, in our hospital, we prefer to place a balloon catheter at night, avoiding expulsion due to daily activity. Thus far, no studies have focused on this issue as a potential mechanism for labour induction. Theoretically, the insertion of a foreign object could increase the risk of intrauterine infections; however, the limited data from our analysis and previous studies did not show any evidence that the cervical ripening balloon catheter contributes to increased infection occurrences [6, 18, 20, 22, 28–31]. More studies are required to address the effects of the balloon-catheters on the rupturing of membranes and infection. In addition, physiologic differences in the mechanism through which balloon catheters induce labour according to parity also must be assessed.

Prior research demonstrated that a Bishop score > 5 was associated with a greater likelihood of vaginal delivery [32, 33]. Although a higher Bishop score was achieved in the double-balloon group in our analysis, there were no differences in the vaginal delivery rates between the two groups. This result interested us, and we hypothesise that there may exist a threshold for the Bishop score that, once achieved, no further effects will be generated; after this threshold is met, the level of hormone secretions takes precedence over cervical conditions. Similar what is observed in our practice, favourable outcomes are rarely observed with balloon usage alone, unless augmentations (e.g., prostaglandin or oxytocin) are utilised.

The larger volume, the application of pressure on two sides (harder expulsion), and the ability to abandon traction when using the double-balloon catheter may explain the observed outcomes. The larger volume balloon may increase the separation between the amniotic membranes and the uterine decidua, resulting in an increase in the local secretion of prostaglandins and enhancing the cervical ripening process. Though 60 ml and 80 ml Foley catheters are more effective than 30 ml catheters [34–36], 80 ml + 80 ml Atad or COOK balloons do not demonstrate superiority to smaller Foley catheters, which may be due to other factors (e.g., traction). We hypothesise that traction may have a greater effect on the induction of labour and that the one-sided application of pressure may interfere with the labour pattern less than two-sided pressure. In theory, traction may cause discomfort for patients. However, this finding has not been confirmed by our analysis. Instead, speculum application prior to catheter insertion, which followed the same procedure in both groups, appears to be the main source of discomfort [15].

Further studies are required to investigate the possible biological mechanisms on cervical ripening and the sources of discomfort, to provide practice guidelines and instrument improvement.

#### Identifying the optimal methods for various populations

Although there were no restrictions on settings, demographics or obstetrics characteristics, the participants from all of the included studies, except for Rab [19], were women with viable singletons and without scarred uteri, making the applicability of our evidence limited. Vaginal birth after caesarean delivery (VBAC) has received increasing attention [37], but identifying the optimal method for labour induction in this specific population remains controversial. Pharmacological methods are often rejected in VBAC women because of greater risks of complications. However, whether balloon catheters can and should be utilised in women with scarred uteri, which manufacturers do not recommend, requires further studies. In addition, twins and other multiple pregnancies are contraindications for the use of balloon catheters, despite the increased frequency of multiple pregnancies. Whether balloon catheters can be used in situations with multiple pregnancies also deserves further study.

### Strengths and limitations

In the current meta-analysis, no demographic or obstetric characteristics were restricted, which increases the applicability of the evidence. We performed evaluations examining evidence of bias and applied quality grades strictly based on the original reports and the Cochrane handbook. The 7 included trials are all rigorous in design, enabling the appraisal and interpretation of their results. Additionally, because bias is more important for studies with subjective events and positive results than for studies with negative results and objective outcomes, such as our analysis, it was acceptable to assume that bias would not practically undermine the results of our analysis.

When extracting data, some outcomes with various forms required data conversions, which likely led to analytical bias. Although we conducted sensitivity analysis specifically to test this possibility, it cannot be clearly determined whether these conversions influenced our outcomes. In addition, the outcomes we chose for this analysis are widely used in practice to avoid potential inconsistencies, and appropriate subgroup analyses were performed to identify potential sources of heterogeneity; however, heterogeneity remained too comprehensive to analyse fully.

The sample size of the current analysis had adequate power for the evaluation of the primary outcome. For some secondary outcomes, fewer data points were available, which may result in insufficient power and higher risks of publication bias. To minimise this bias and to involve more relevant studies, we have done our best to search databases using a wide range of publication years, to consider potentially eligible reviews and to fully utilise trial registration databases, with sensitivity-maximising search filters. Unfortunately, we are still incapable of accessing conference abstracts or proceedings and grey literature. Thus, publication bias cannot be excluded completely, and caution should be taken.

The procedures performed during our analysis to reduce bias and assess risks can provide direction for further research, although not all of these are necessary.

## Conclusions

Both kinds of balloon catheter perform similarly with regards to efficacy, efficiency, safety and patient satisfaction. The single-balloon device appears to be more economical and practical, particularly in low resource settings.

## Data Availability

All data generated or analysed during the current study are available from the corresponding author on reasonable request.

## Abbreviations

CCRB: Cook Cervical Ripening Balloon
CDSR: Cochrane Database of Systematic Reviews
CENTRAL: Cochrane Central Register of Controlled Trials
CIs: Confidence intervals
MDs: Mean differences
PICOS: Population, intervention, comparison, outcomes and study designs
PROs: Patient-reported outcomes
RCTs: Randomised controlled trials
RRs: Relative risks
USFDA: United States Food and Drug Administration
VAS: Visual analogue scale
VBAC: Vaginal birth after caesarean delivery
